# Closing the knowledge gap in pelvic neuroanatomy: assessment of a cadaveric training program

**DOI:** 10.1186/s12909-020-02443-4

**Published:** 2021-01-07

**Authors:** Ioana Marcu, Adrian Balica, Jeffrey A. Gavard, Eugen C. Campian, Gustavo Leme Fernandes, M. Jonathon Solnik, Vadim Morozov, Nucelio Lemos

**Affiliations:** 1 Department of Obstetrics and Gynecology, Saint Louis University School of Medicine, Saint Louis, MO USA; 2 Department of Obstetrics and Gynecology, Rutgers Robert Wood Johnson Medical School, New Brunswick, NJ USA; 3 Department of Obstetrics and Gynecology, Faculdade de Medicina do ABC, Santo André, São Paulo, Brazil; 4 Department of Obstetrics and Gynecology, Division of Gynecology and Minimally Invasive Surgery, Mount Sinai Hospital, Toronto, ON Canada; 5 Department of Obstetrics and Gynecology, Division of Minimally Invasive Gynecologic Surgery, University of Maryland, Baltimore, MD USA; 6 Department of Obstetrics and Gynecology, University of Toronto, Toronto, ON Canada

**Keywords:** Cadaver, Dissection, Neuroanatomy: neuropelviology

## Abstract

**Background:**

The objective of this study is to characterize participants in a laparoscopic cadaveric neuroanatomy course and assess knowledge of pelvic neuroanatomy before and after this course.

**Methods:**

This is a survey-based cohort study with a setting in a university educational facility. The participants are surgeons in a multiday laparoscopic cadaveric pelvic neuroanatomy course. Participants completed a precourse survey, including demographics and comfort with laparoscopic surgery. They then completed an identical precourse and postcourse anatomic knowledge test. Main outcomes are scores on the anatomic knowledge test precourse and postcourse.

**Results:**

44 respondents were included: 25 completed fellowship, 15 completed residency, 2 were residents, and 2 were fellows. Participants were on average 11.09 years post training, with an average of 8.67 years from training if they completed fellowship and 18.62 years if they completed residency only. 22 of 42 respondents strongly agreed or agreed they are comfortable performing complex laparoscopic hysterectomies. The average precourse score was 32.18/50 points and the mean difference score (MDS, defined as mean of Postcourse scores minus Precourse scores) was 9.80, showing significant improvement (*p* <  0.001). Precourse and MDS scores were not significantly different when comparing country of practice, level of training, or time since training.

**Conclusion:**

Baseline knowledge of pelvic neuroanatomy was similar among groups when comparing fellowship status, place of training, or time since training. There was significant improvement in knowledge after training in this dissection method. This course garnered interest from surgeons with broad training backgrounds.

## Capsule

Neuropelviology can be taught through a standardized and reproducible dissection protocol.

## Background

While female pelvic anatomy is already complex in the setting of normal anatomic relationships, endometriosis, previous surgery, or neoplasms and other conditions can distort normal anatomy, making the understanding of the region more complex. Therefore, a comprehensive and thorough understanding of pelvic anatomy is crucial for successfully managing these conditions [1–4].

As our understanding of functional and surgical pelvic anatomy evolves, there has been a recent trend towards post-graduate laparoscopic training courses and mini-residencies [5]. These courses address training of normal anatomy and also topics such as surgical complications, which cannot be routinely practiced in live patients [6], hysterectomies, lymph node biopsies [7], and dissection of neurovascular bundles [8].

As the role of laparoscopic surgery in residency/fellowship training continues to evolve and as the field of neuropelveology develops, ongoing studies of post-graduate courses with a focus on pelvic neuroanatomy are necessary. The purpose of our study was to introduce a method of retroperitoneal pelvic nerve dissection as well as assess baseline anatomic knowledge and improvement as a result of the course. We also characterized participants in the postgraduate cadaveric laparoscopic course on pelvic neuroanatomy.

## Methods

Participants in the laparoscopic course were recruited from the United States and internationally through direct communication, electronic means of advertisement, and mail-in brochures. Participants in this study were recruited from those participating in the course. All participants in the course were age 18 or greater, resident physicians, fellows in training, or physicians out of training. Participants in the course were from both North and South America, due to the fact that the course instructors were from North America and areas of South American, mainly Brazil.

Course instructors consisted of Minimally Invasive Gynecologic Surgeons and Female Pelvic Medicine and Reconstructive Surgeons who specialize in nerve sparing procedures. All course instructors were fluent in English.

Participants were approached prior to the start of the course for participation in a quality improvement project. The initial data were collected as a quality improvement project. IRB approval was subsequently obtained for retrospective analysis of the collected data to be used as research.

Primary study objectives were to introduce a systematic method of cadaveric laparoscopic dissection of pelvic nerves, to assess baseline anatomic knowledge of course participants, and to assess change in Anatomic Survey scores after the course.

Secondary objectives were to describe course participants characteristics/demographics, expectations, comfort level with laparoscopy, and satisfaction after the course as well as to perform sub-analyses that assess baseline anatomic knowledge of course participants and change in the Anatomic Survey scores after the course.

Inclusion criteria for the study were any participant data sets in the quality improvement project with completed Precourse and Postcourse Anatomy Surveys. Exclusion criteria were either uncompleted Precourse or Postcourse Anatomy Surveys. Given that the method of recruitment was of all those participating in the cadaveric course, there were no identifiable sources of bias in recruitment for the study that are not inherent to the course.

Female specimens with intact abdominal cavity were prepared for this course, using the ‘soft’ or Thiel’s method, which allows for insufflation and use of laparoscopic technique.

Course duration was 2 days. The course consisted of morning lectures of pelvic anatomy and narrated videos of standardized step by step dissection. All participants were aware that training would be in English and therefore participants were self-selected to be comfortable with presentations in English. This method represented a new model of pelvic dissection involving the somatic and autonomic nerves in a reproducible, step-wise fashion.

In the afternoon session, participants performed laparoscopic dissections as taught in the morning session. Self-assigned pairs worked on the same cadaver and remained paired for the entirety of the course. Course faculty were available during dissection reinforcing the steps of anatomic dissection and anatomic knowledge by answering questions and guiding the participants.

There were no sources of funding for this study.

**Dissection steps are described below:**

The following trocars are placed: one umbilical, two lateral and one suprapubic.

**Fig. 1:**
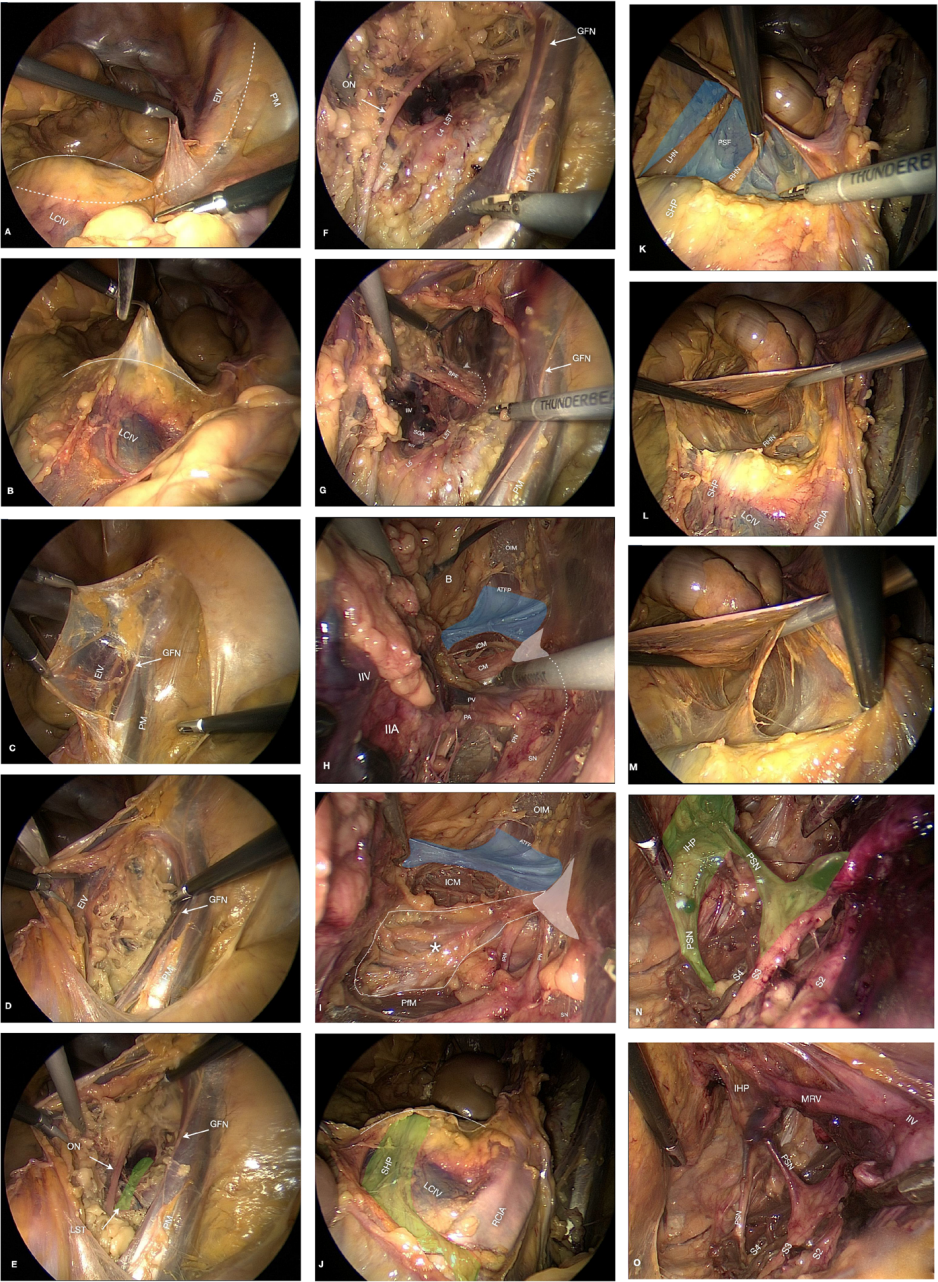
**a**. LCIV: Left common Iliac Vein. EIV: External Iliac Vein. PM: Psoas Muscle. Solid Line: Sacral Promontory. Dashed Line: incision. **b**. LCIV: Left Common Iliac Vein. Solid Line: Sacral Promontory. **c**. EIV: External Iliac Vein. Arrow GNF: Genitofemoral Nerve. PM: Psoas Muscle. **d**. Opening Obturator Fossa. Arrow GNF: Genitofemoral Nerve. EIV: External Iliac Vein. **e**. Within Obturator Fossa. Arrow ON: Obturator Nerve. Arrow LST to Green Shaded Area: Lumbosacral Trunk. Arrow GNF: Genitofemoral Nerve. PM: Psoas Muscle. **f**. Arrow ON: Obturator Nerve. L4: Lumbar Nerve Root. LST: Lumbosacral Trunk. Arrow GNF: Genitofemoral Nerve. PM: Psoas Muscle. **g**. IIV: Internal Iliac Vein. SGV: Superior Gluteal Vein. SVA: Superior Gluteal Artery. L4, L5: Lumbar Nerve Roots. LST: Lumbosacral Trunk. SPF: Sciatic Perineural Fat. White Shaded Arrow Head: Ischial Spine. Dotted line: Sciatic Notch. GFN: Genitofemoral Nerve. PM: Psoas Muscle. **h**. IIV: Internal Iliac Vein. IIA: Internal Iliac Artery. PV: Pudendal Vein. PA: Pudendal Artery. PN: Pudendal Nerve. ICM: Iliococcygeus Muscle. Red Shaded Area CM: Coccygeus Muscle. White Shaded Area: Ischial Spine. Asterix: Sacrospinous ligament. ATFP: Arcus Tendineus Fascia Pelvis. Blue Shaded Area: Endopelvic fascia. OIM: Obturator internus muscle. SN: Sciatic Nerve. Dotted Line: Sciatic Notch. **i**. ICM: Iliococcygeus Muscle. White Shaded Area: Ischial Spine. Asterix on Area Outlined with White Line: Sacrospinous Ligament. PfM: Piriformis Muscle. ATFP: Arcus Tendineus Fascia Pelvis. Blue Shaded Area: Endopelvic fascia. IRN: Inferior Rectal Nerve. PV: Pudendal Nerve. OIM: Obturator internus muscle. SN: Sciatic Nerve. **j**. Solid Line: Sacral Promontory. Green Shaded Area SPH: Superior Hypogastric Plexus. LCIV: Left Common Iliac Vein. RCIA: Right Common Iliac Artery. **k**. SHP: Superior Hypogastric Plexus. LHN: Left Hypogastric Nerve. RHN: Right Hypogastric Nerve. Blue shaded area PSF: Presacral Fascia. **l**. SHP: Superior hypogastric plexus. RHN: Right Hypogastric Nerve. LCIV: Left Common Iliac Vein. RCIA: Right Common Iliac Artery. U: Ureter. **m**. Overview of Presacral Space. **n**. Green Shaded Area IHP: Inferior Hypogastric Plexus. Green Shaded Area PSN: Pelvic Splanchnic Nerve. S2, S3, S4: Sacral Nerve Roots. **o**. IHP: Inferior Hypogastric Plexus. MRV: Middle Rectal Vein. IIV: Internal Iliac Vein. PSN: Pelvic Splanchnic Nerve. S2, S3, S4: Sacral Nerve Roots

The attendant standing on the right of the specimen will start acting as surgeon and the one standing on the left as the assistant. They proceed as follows:

1. Incise the peritoneum on top of the right psoas muscle tendon and extend the incision to the promontory (Fig. 1a, b, c);
2. Identify and lateralize the genitofemoral nerve and bluntly dissect the space between the medial border of the psoas muscle and the external iliac vessels to develop the iliolumbar and obturator spaces on the right side (Fig. 1c, d, e);
3. Identify the obturator nerve and, just posteriorly to it, the lumbosacral trunk and the sciatic nerve (Fig. 1e, f, g);
4. Separate the perineural fat at the sciatic notch and expose the superior and inferior gluteal nerves (Fig. 1g, h);
5. Carry the dissection further distally and identify the endopelvic fascia, the arcus tendineus fascia pelvis and the levator ani muscles, as well as the ischial spine, the sacrospinous ligament and the pudendal nerve crossing underneath it (Fig. 1h, i);
6. Re-focus dissection at level of the sacral promontory; dissect the presacral fascia from the pararectal peritoneum and identify the superior hypogastric plexus and the hypogastric nerves (Fig. 1j, k);
7. Incise the presacral fascia laterally to the right hypogastric nerve and develop the presacral space down to the coccyx and laterally to the hypogastric fasciae bilaterally (Fig. 1l, m);
8. Incise the hypogastric fascia and identify the piriformis muscle, the sacral nerve roots lying on its ventral surface and the pelvic splanchnic nerves branching out of them and running towards the inferior hypogastric plexus (Fig. 1n, o).

At this point, the attendant standing on the left of the specimen assumes the surgeon role, giving the camera to the assistant on the right. The next steps are:

1. Incise the presacral fascia laterally to the left hypogastric nerve;
2. Incise the left hypogastric fascia and identify the piriformis muscle, the sacral nerve roots lying on its ventral surface and the pelvic splanchnic nerves branching out of them and running towards the inferior hypogastric plexus;
3. Incise the peritoneum on top of the left psoas muscle tendon;
4. Identify and lateralize the genitofemoral nerve and bluntly dissect the space between the medial border of the psoas muscle and the external iliac vessels to develop the iliolumbar and obturator spaces on the left side;
5. Identify the obturator nerve and, just posteriorly to it, the lumbosacral trunk and the sciatic nerve;
6. Separate the perineural fat at the sciatic notch and expose the superior and inferior gluteal nerves;
7. Carry the dissection further distally and identify the endopelvic fascia, the arcus tendinous fascia pelvis and the levator ani muscles, as well as the ischial spine, the sacrospinous ligament and the pudendal nerve crossing underneath it;
8. Re-focus the dissection on the lateral aspect of the psoas muscle, incise the fascia transversalis and identify the lateral femoral cutaneous nerve and the femoral nerves;
9. Extend the dissection proximal and identify the ilioinguinal and iliohypogastric nerves.

The attendant standing on the right of the specimen reassumes the surgeon role and performs steps on the right side.

Participants were asked to complete the following questionnaires:

Before the course, participants filled out a survey to assess demographics, level of education, prior laparoscopic training, and a Precourse Subjective Survey to assess level of comfort with laparoscopic surgery, and expectations for the course. Participants were alsoasked to complete a multiple-choice test of anatomic knowledge (Precourse Anatomy Survey) before the first didactic lecture.

Postcourse, participants filled out a multiple-choice test of anatomic knowledge identical to the Precourse Anatomy Survey (Postcourse Anatomy Survey) and a subjective survey regarding satisfaction with the course.

**Fig. 2:**
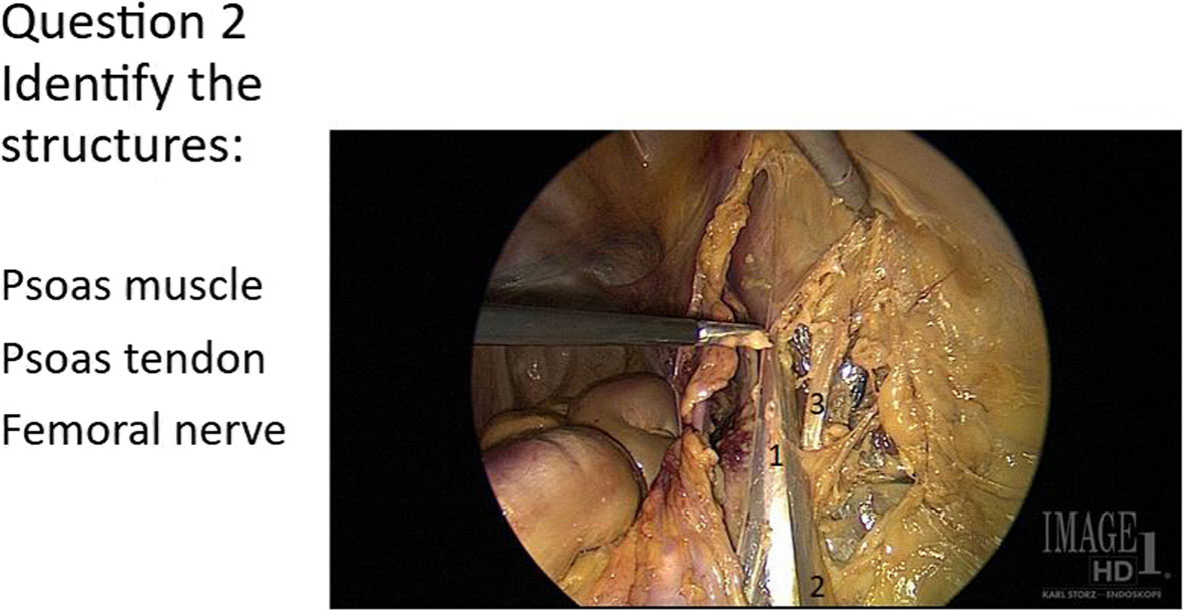
Sample test question. This is a sample of a “matching” test question to identify laparoscopic structures

The identical Precourse and Postcourse Anatomy Surveys consisted of 12 images in which participants were asked to match structures on printed photograms from cadaveric laparoscopic dissections with answer choices, with 50 structures for a maximum score of 50 points. The tests were picture-based and the only English words used were anatomic terms. A representative sample question is included (Fig. 2). Given that all South American participants had registered for the course knowing it would be in English and thus self-selected for English proficiency, and given that the test was picture-based, we did not expect language to present a barrier to understanding the test.

We analyzed the data to determine demographics of the participants in the study. We characterized participants’ expectations and analyzed how those expectations were met. We assessed baseline anatomic knowledge and differences in the anatomic knowledge before the course and after course using paired t-tests if the data were normally distributed and the Wilcoxon Signed Rank test if the data were not normally distributed. Differences in Precourse Anatomy Survey Scores or Deviation scores (Postcourse Anatomy Survey score- Precourse Anatomy Survey score) for different sub-analysis groups were using independent Student’s t-test or the Mann Whitney U test, depending on distribution normality. Normality was assessed using the Shapiro- Wilk test. Cronbach alpha test was used to asses for internal consistency of subjective survey questions. Data were analyzed using SPSS Version 23.0 for Windows (IBM, Armonk, New York). A *P*-values < 0.05 was used to denote statistical signficiance.

## Results

Participants were included in the analysis if they filled out both Precourse and Postcourse Anatomy Surveys, irrespective of completion status of other surveys. A total of 44 participants were included in the analysis. There were 18 participants from North America included in the session, and 26 from South America. The mean age of participants was 43.4 years. Of the 44 participants, 25 completed fellowship, 15 completed residency only, 2 were current residents, and 2 were current fellows. When pooling all data sets, participants were 11.09 years post-training, as defined by time elapsed since residency or fellowship. Participants averaged 8.67 years from training if they completed a gynecologic fellowship and 18.62 years from training if they completed residency only (Table 1).

**Table 1:** Characteristics of Course Participants

	**Number**	
**All**	44	
**Completed Residency only**	15	
**Completed Fellowship**	25	
**Current Residents**	2	
**Current Fellows**	2	
	**Number**	**Age (years)**
**All**^**a**^	43	43.42 (10.17)
**Completed residency only**^**b**^	14	48.21 (10.16)
**Fellowship trained**	25	42.76 (8.57)
**North American**	18	42.61 (10.05)
**South American**^**c**^	15	44.00 (11.19)
	**Number**	**Time since training (years)**
**All**^**d**^	37	11.09 (10.04)
**Completed residency only**^**e**^	13	18.62 (11.24)
**Fellowship trained**^**f**^	24	8.67 (8.68)
**North American**^**g**^	17	10.35 (8.68)
**South American**^**h**^	20	13.70 (11.93)

Age presented as Mean (Standard Deviation). Years from training represented as Mean, Median (Standard Deviation)

^a^Age unknown for one participant

^b^Data known for 14 of 15 possible participants

^c^Data known for 15 of 16 possible participants

^d^Data presented for 37 of 44 total participants, as 2 are current residents, 2 current fellows, 3 unknown

^e^Data known for 13 of 15 possible participants

^f^Data known for 24 of 25 possible participants

^g^Data presented for 17 of 18 possible participants as one is an active fellow

^h^Data presented for 20 of 26 possible participants, as 2 are active fellows, 1 an active resident, and 3 unknown

When asked if their training program prepared them for a laparoscopic hysterectomy, 13 of 43 (30.2%) strongly agreed or agreed. Of 42 participants 35 (83.3%) strongly agreed or agreed that they felt comfortable performing less complicated laparoscopic surgeries such as bilateral tubal ligation. Thirty-four of 42 (81%) strongly agreed or agreed they felt comfortable performing complex surgeries such as hysterectomies in uncomplicated cases, while 22 of 42 (52.4%) strongly agreed or agreed they felt comfortable performing complex laparoscopic surgeries such as hysterectomies in complicated cases.

Of 43 participants, 27 (62.8%) responded that they had taken courses like this one in the past.. When asked if similar courses impacted practice immediately after completion, 19 of 25 (76%) respondents strongly agreed and the remaining 6 of 25 (24%) agreed. No participants were neutral, disagreed, or strongly disagreed. When asked if similar courses impacted long term practices, 17 of 24 (71%) who responded strongly agreed, 6 of 24 (25%) agreed, and 1 of 24 (4%) were neutral. No respondents disagreed or strongly disagreed.

The mean Precourse Anatomy Survey score for all participants was 32.18 + 8.30 and the mean difference score (MDS, defined as mean of Postcourse Anatomy Survey scores minus Precourse Anatomy Survey scores) was 9.80 + 8.46, showing significant improvement between Precourse and Postcourse Anatomy scores (*p* <  0.001.) The mean Precourse Anatomy score for North Americans (*N* = 18) was 31.00 + 7.56 and the MDS was 10.22 + 6.15. The mean Precourse Anatomy score for South Americans (*N* = 26) was 33.00 + 8.83 and theMDS was 9.50 + 9.86. The difference between mean difference scores was non-significant *p* = 0.77 (Table 2). Both groups improved, but they did not differ significantly in their degree of improvement.

**Table 2:** Precourse Anatomy Survery Scores, Postcourse Anatomy Survey Scores and Mean Deviation Scores

	Precourse score	Postcourse score	Mean Deviation Score	*p*-Value
All (*N* = 44)	32.18 (8.30)	41.98 (6.32)	9.80 (8.46)	< 0.001 between precourse and postcourse score*
North American (*N* = 18)	31.00 (7.56)	41.22 (6.54)	10.22 (6.15)	< 0.001
South American (*N* = 26)	33.00 (8.83)	42.50 (6.23)	9.50 (9.86)	< 0.001
*p*-value 0.44 for Precourse scores, 0.77 for MDS				
Previously taken a similar course (*N* = 27)^a^	33.33 (8.58)	42.85 (6.32)	9.52 (8.47)	< 0.001
Not previously taken a similar course (*N* = 16)^a^	29.69 (7.47)	40.56 (6.45)	10.88 (8,55)	< 0.001
*p*-value 0.17 for Precourse scores, 0.62 for MDS				
Fellowship trained (*N* = 25)^b^	32.32 (7.16)	43.40 (5.42)	11.08 (7.15)	< 0.001
Residency completed only (*N* = 15)^b^	31.07 (10.08)	38.93 (6.76)	7.87 (10.08)	< 0.01
*p*-value 0.65 for Precourse scores, 0.25 for MDS				
< 5 years since training (*N* = 12)^c^	32.50 (8.69)	43.25 (6.74)	10.75 (8.51)	< 0.001
> 5 years since training (*N* = 25)^c^	30.84 (8.37)	41.00 (5.99)	10.16 (8.19)	< 0.001
*p*-value 0.58 for Precourse scores, 0.84 for MDS				
< 5 years since training, completed fellowship (*N* = 11)^d^	33.09 (8.86)	44.00 (6.53)	10.91 (8.91)	< 0.01
> 5 years since training, completed fellowship (*N* = 13)^d^	31.00 (5.34)	43.08 (4.73)	12.08 (4.97)	< 0.001
*p*-value 0.48 for Precourse scores, 0.70 for MDS				
< 5 years since training, completed residency only (*N* = 1)^e^	26.00	35.00	9.00	–
> 5 years since training, completed residency only (*N* = 12)^e^	30.67 (11.03)	38.75 (6.58)	8.08 (10.51)	< 0.05
*p*-value 0.69 for Precourse scores, 0.94 for MDS				
Age < 43 years (*N* = 23)^f^	32.39 (7.61)	42.43 (6.87)	10.04 (7.92)	< 0.001
Age > 43 years (*N* = 20)^f^	32.05 (9.41)	41.20 (5.77)	9.15 (9.29)	< 0.001
*p*-value 0.90 for Precourse scores, 0.74 for MDS				
Age < 43 years, completed fellowship (*N* = 13)	31.92 (7.72)	43.62 (6.37)	11.69 (7.74)	< 0.001
Age > 43 years, completed fellowship (*N* = 12)	32.75 (6.82)	43.17 (4.45)	10.42 (6.72)	< 0.001
*p*-value 0.78 for Precourse scores, 0.67 for MDS				
Age < 43 years, completed residency only (*N* = 6)^g^	31.33 (7.31)	38.50 (7.34)	7.17 (6.94)	0.05
Age > 43 year, completed residency only (*N* = 8)^g^	31.00 (12.84)	38.25 (6.54)	7.25 (12.50)	0.15
*p*-value 0.95 for Precourse scores, 0.99 for MDS				

All scores represented as Mean (Standard deviation)

N varies due to number of respondents for each category

^a^Status of having taken previously similar courses unknown for one participant

^b^4 participants were active residents or fellows

^c^Years since training is not applicable to 2 current residents and 2 current fellows and was unknown for 3 participants

^d^Years since training was unknown for 1 participant

^e^Years since training was unknown for 2 participants

^f^Age unknown for 1 participant

^g^Age unknown for 1 participant

Having participated in similar courses in the past did not significantly impact Precourse scores or MDS scores. The mean Precourse Anatomy score for the 27 who had taken similar courses in the past was 33.33 + 8.58 and 29.69 + 7.47 for those who had not taken similar courses in the past (*p* = 0.17). There was statistically significant improvement in participants who had and who had not taken similar courses in the past (Table 2).

Comfort level with performing laparoscopic surgeries did not impact Precourse Anatomy scores. Twenty two of 42 (52.4%) participants strongly agreed or agreed that they felt comfortable performing complex laparoscopic surgeries, such as hysterectomies. Twenty of 42 (47.6%) participants were either neutral, disagreed, or strongly disagreed when they were asked the same question. The difference between the mean Precourse Anatomy scores of those who strongly agreed or agreed with the statement (32.00 + 7.70) and those who were neutral, disagreed, or strongly disagreed (31.85 + 9.28) was not significantly different (*p* = 0.96, Table 3). There was also no significant difference between the Precourse scores of participants who strongly agreed or agreed they felt comfortable performing less complicated laparoscopic surgeries such as bilateral tubal ligation (Precourse score 32.29 + 7.98) and those who were neutral, disagreed, or strongly disagreed with the statement (mean Precourse score 30.14 + 10.70 *p* = 0.54,Table 3).

**Table 3:** Surgical Comfort Level and Precourse Anatomy Survery Scores

N total = 42	Precourse score for those who Strongly Agreed or Agreed	Precourse score for those who are Neutral, Disagreed, or Strongly Disagreed	*p*-value
“I feel comfortable performing less complicated laparoscopic surgeries such as bilateral tubal ligation”	32.29 (7.98) *N* = 35	30.14 (10.70) *N* = 7	0.54
“I feel comfortable performing complex laparoscopic surgeries such as hysterectomies in uncomplicated cases”	31.82 (8.40) *N* = 34	32.38 (8.83) *N* = 8	0.87
“I feel comfortable performing complex laparoscopic surgeries such as hysterectomies in complicated cases”	32.00 (7.70) *N* = 22	31.85 (9.28) *N* = 20	0.96

Participants were provided the statements in column 1 prior to starting the course. They were asked to answer on a 5 point scale from strongly agree (1) to strongly disagree (5). Those who answered strongly agreed or agreed were grouped together. Those who answered neutral, disagreed, or strongly disagreed were grouped together. Precourse scores of the groups were compared. All scores represented as Mean (Standard deviation). Data presented as Precourse score (Standard Deviation) with *N* = number of respondents out of 44

The mean Precourse Anatomy score for persons who completed gynecological fellowship (*N* = 25) was 32.32 + 7.16 and the MDS was 11.08 + 7.15. The Precourse Anatomy score for those who completed residency only (*N* = 15) was 31.07 + 10.08 and the MDS was 7.87 + 10.08 There was no statistical difference in the MDS (*p* = 0.25) between those who completed fellowship and those who completed residency only (Table 2).

We found that having finished training (as defined as fellowship or as residency) within 5 years did not lead to significant difference in either the Precourse Anatomy scoresor the MDS. When selecting for only those who completed fellowship, or those who completed only residency, the mean Precourse Anatomy score and the MDS were not significantly different between those who had completed their respective training within 5 years or more than 5 years (Table 2).

The same analysis was carried out comparing the Precourse Anatomy score and the MDS for those above the age of 42 and those below the age of 42, given that 42 was the median age. The analysis was performed for all participants, and then only for those who had completed fellowship and those who had completed residency only, and no statistical differences were found between these groups (Table 2).

Satisfaction was high for this course. Thirty-one of 44 (70%) were very satisfied, 12 of 44 (27%) were satisfied and only 1 of 44 (2%) was very dissatisfied. When asked to rate how integral the following components of the course were, on a scale from 1 to 5, with 1 being most integral and 5 being least integral, participants gave the cadaveric component a rating of 1.14, followed by the video rating of 1.32, and the lecture component 1.57.

Participants were also asked to rate perceived improvements as a result of this course, on a scale from 1 to 5 with 1 being strongly agreed and 5 being strongly disagreed. An average rating of 1.30 was given by 44 participants when they were asked if they believed this course improved knowledge of pelvic neuroanatomy (Table 4). An average rating of 1.64, 1.75, and 1.80 were given when participants were asked if this course improved their knowledge of pelvic vasculature, pelvic musculature, and knowledge of the ureter anatomy, respectively. An average rating of 1.89 was given when participants were asked if they believed this course improved laparoscopic dissection method and 2.05 when asked if the course improved surgical skills.

**Table 4:** Subjective Improvements After This Course

	Average of 1–5 Scores	1 = Strongly Agreed	2 = Agreed	3 = Neutral	4 = Disagreed	5 = Strongly Disagreed
Improved my surgical skills	2.05 (0.95) *N* = 44	13	21	6	3	1
Improved my laparoscopic dissection method	1.89 (0.91) *N* = 44	17	18	7	1	1
Improved my knowledge of neuroanatomy	1.30 (0.69) *N* = 44	34	9	0	0	1
Improved my knowledge of pelvic vasculature	1.64 (0.85) *N* = 44	23	17	2	1	1
Improved my knowledge of pelvic musculature	1.75 (0.75) *N* = 44	19	20	3	1	1
Improved my knowledge of pelvic ureter	1.80 (0.89) *N* = 44	19	18	5	1	1

Participants were provided the statement “This course... improved my surgical skills,” and so on for all of the statements in column one. They were asked to answer on a 5 point scale from strongly agreed (1) to strongly disagreed (5). All scores in column 2 represented as Mean (Standard deviation), *N* = number of respondents out of 44

Nineteen of 44 (43.2%) participants strongly agreed and 21 of 44 (47.7%) agreed that this course would change the way the practiced laparoscopic surgery. Twenty-three of 44 (52.3%) participants strongly agreed and 19 of 44 (43.2%) agreed that they would take a similar course in the future. Sixteen of 44 (36.4%) strongly agreed and 20 of 44 (45.5%) agreed that they would take the same course again.

We assessed internal consistency of the subjective surveys through the use of Cronbach alpha testing. For 7 questions in the Precourse subjective survey that assessed course expectations, the Cronbach alpha score was 0.890, corresponding to a survey with moderate-high internal consistency. For the 7 questions in the Postcourse subjective survey assessing whether the course resulted in improved knowledge/skills, the Cronbach alpha score was 0.933, corresponding to high internal consistency in that survey. The Cronbach alpha of the three questions in the Precourse Subjective Survey which interrogate the comfort level of the participants with laparoscopic procedures of increasing complexity is 0.671, which represents an acceptable level of internal consistency.

In regards to the anatomic surveys, the 12 Precourse Anatomy Survey questions have a Cronbach Alpha at 0.728 and the identical 12 Postcourse Anatomy Survey questions have a Cronbach Alpha of 0.685, corresponding to good and acceptable internal consistency, respectively.

## Conclusion

Since the 1990s, the development of video-laparoscopy, MRI, surgical instrumentation, and operative techniques have truly revolutionized knowledge of pelvic anatomy. This has allowed for the development of new procedures and improvement of outcomes in virtually all pelvic procedures.

Neuropelveology is one illustrative example of those advances in knowledge. It is an emerging field [9, 10]. opened by the advances of laparoscopic technique and instrumentation, which allowed for the development of the Laparoscopic Neuronavigation (LANN) technique. This technique revolutionized our knowledge of pelvic neuroanatomy and the pathology of pelvic nerves, and initiated the movement of nerve-sparing procedures in radical pelvic surgery [11–16].

These fast advances in medical knowledge, however, have created a knowledge gap. Given the newness of the field, neuropelveology has not been taught as part of most medical school or residency education. Moreover, it is an area of knowledge that does not lend itself to typical residency anatomic education in departments without neuropelveologists.

Most surgical anatomic teaching during residency is ‘hands on” in the operating room or based on visual media of anatomy [1]. Cadaveric dissection training has been shown to improve laparoscopic surgical skills and improve anatomic knowledge at both the resident and fellow training level [8, 17–20]. This program was developed by minimally invasive gynecologists and urogynecologists who perform radical nerve sparing procedures, such as removal of deep infiltrating endometriosis from sacral nerve roots and who identified a neuroanatomy knowledge gap in their trainees. Given the high skill level and specific indications required to perform this type of procedure on a live patient, cadaveric training was thought to be the safest, most thorough training method. This training course was developed in this context.

Baseline anatomic knowledge has been assessed after training in other studies; after a surgically-intensive fellowship such as gynecologic oncology, there was inadequate knowledge of anatomic structures [8]. Resident physicians lose a considerable amount of basic anatomic knowledge while transitioning from student to clinician [21, 22]. In our group, there was statistically significant improvement in the pelvic neuroanatomic knowledge after the course. Thw objective improvement on Anatomy Survey is supported by participant report of subjective improvement.

There was no significant difference in the Precourse Anatomy scores or in the MDS when subcategories of participants were compared. The similar Precourse Anatomy scores may be due to a similar baseline neuropelviology knowledge among OBGYNs, even those with different surgical comfort levels. However, this may also be due to self-selection of participants who feel they are deficient in this subject matter. It must be noted that this is in the context of a non-validated questionnaire and that lack of statistical difference may be due to the lack of power to identify such differences. That being said, we did find statistically significant differences in Precourse and Postcourse Anatomy scores, indicating that our study was powered to detect difference in scores.

We identified a trend, although not statistically significant, where those who completed fellowship had a higher MDS compared to those who did not complete fellowship. Their Precourse Anatomy scores were nearly identical, which would indicate similar baseline neuroanatomy knowledge . The trend of greater improvement in those who completed fellowship may be due to fellowship-trained participants having more training and/or exposure to complex procedures and are, therefore, more prepared to assimilate complex anatomic training. This finding further reflects the previously mentioned knowledge gap even in fellowship-trained surgeons, and reinforces the utility of these cadaver courses.

We also identified a trend whereby the Precourse Anatomy scores of participants who had participated in similar courses in the past were higher than those without previous participation in similar courses. Although not statistically significant, it does beg the question of long-term retention of neuropelviology from courses such as this, which is as of yet unknown.

Concurring with other studies assessing satisfaction after laparoscopic courses [7], this course had high satisfaction, with 43 of 44 participants being either very satisfied or satisfied with the course and with the cadaveric component being considered the most integral part.

Reflecting similar results in other studies, participants believe that this course will change their practice [6, 19, 23, 24]. Our participants also overwhelmingly agreed similar past courses impacted both their short term and longer-term practice patterns. 95% of participants strongly agreed or agreed that they would participate in a similar course in the future and that 81% would participate in the same course in the future. Participants also expected that improved anatomic knowledge would translate to greater surgical skill.

Limitations of this study include that the anatomy test is not validated. However, there is no validated testing tool for pelvic neuroanatomy. Another aspect of the anatomy test to consider is that the questions in the Precourse Anatomy Survey and Postcourse Anatomy Survey were identical, and that exposure to the same questions twice might itself lead to improved scores. That being said, pictures were selected to highlight important steps of the dissection at the angle and distance that the laparoscopist would naturally encounter the anatomy. Moreover, we considered that a 2-day latency between the Anatomy Surveys would provide enough time between tests so that the memory of the first test would no longer be in short term recall. These two factors played into our decision to administer identical surveys. The Cronbach Alpha scores of the Precourse and Postcourse Anatomy Surveys are 0.728 and 0.685, respectively. These scores both reflect an acceptable level of internal consistency. However, a somewhat lower Cronbach Alpha score in the setting of an educational tool may be a reflection of heterogeneous gaps in training or knowledge rather than an inconsistent survey tool.

This study was also limited by lack of a control group, a group that does not undergo the cadaveric training and dissection. However, given that the participants in the study were registered participants in the course, there was no option for a control group. Therefore, while our study indicates that participants learned pelvic neuroanatomy, we cannot conclude the superiority of this method as compared to a non-cadaveric method. The purpose in assessing Precourse and Postcourse Anatomy scores was to ensure improvement with the course, not to compare methods of teaching pelvic neuroanatomy. In regards to the other survey questions, such as regarding comfort level with various surgeries, they suffer from the intrinsic weakness of recall bias.

We have noted that over half of the participants do not speak English as a primary language. However, the Precourse Anatomy scores were not significantly different between participants from North America compared to those from South America, which supports that language was not a barrier to understanding the lectures or the tests.

Strengths of this study include a robust description of a reproducible retroperitoneal dissection method for identification of neuroanatomy that references landmarks that are approachable to both fellowship-trained and generalist OBGYNs. Given that participants in this study come from a diverse geographic background and at different levels of education, we believe that this study is generalizable to a broad cross section of gynecologic surgeons, especially as most gynecologic surgeons do not have significant background in pelvic neuroanatomy.

Future directions of study would include targeting this course towards specific subspecialties, with the dissection course and anatomic landmarks tailored towards subspecialty procedures. Another possible future direction would be a web-based course, focusing on a recording of the dissection. While this would not allow for the three-dimensional feel provided by performing the procedure oneself, it would provide academic benefit. Given the need for education on pelvic neuroanatomy, another future avenue of study would be to validate the anatomic survey. A version of this course, which teaches, but does not focus on neuroanatomy, is currently being taught to OB/GYN resident physicians at our institution, as a result of positive feedback from this course.

In conclusion, laparoscopic pelvic neuroanatomy is an unfamiliar subject to both intermediate and advanced gynecologic surgeons. However, it can be effectively taught through a standardizeddissection protocol. The development and reproduction of such courses is essential for closing the neuroanatomic knowledge gap in and may prove important for reducing perioperative complications of patients undergoing pelvic surgery.

## Data Availability

All original data and master lists have been destroyed in the appropriate manner. Data sets exist in de-identified form in a RedCap database. Datasets used are available from the corresponding authors on reasonable request.

## Abbreviations

MDS: Mean difference score, defined as mean of Postcourse Anatomy Survey scores minus Precourse Anatomy Survey scores
